# Gene regulation analysis of the effects of evodiamine on tongue squamous cell carcinoma

**DOI:** 10.1002/jcb.28869

**Published:** 2019-05-12

**Authors:** Yuyan Wu, Jing Wang, Jiayuan Zhao, Yunxia Zhang, Yunjie Sun, Jian Chen, Jing Wang

**Affiliations:** ^1^ Department of Periodontology School/Hospital of Stomatology, Lanzhou University Lanzhou Gansu China; ^2^ Department of Pediatric Surgery The First Hospital of Lznzhou University Lanzhou Gansu China

**Keywords:** bioinformatics analysis, evodiamine, molecular mechanisms, tongue squamous cell carcinoma

## Abstract

**Objective:**

To use gene chip technology to study the effects of evodiamine (EVO) on the gene expression profile of tongue squamous cell carcinoma (TSCC) CAL‐27 cell line, for the purpose of analyzing the mechanisms underlying the effects of EVO on gene expression and functional regulation of TSCC cells at the gene level.

**Method:**

Differentially expressed genes in CAL‐27 cells treated with EVO were detected using gene chip technology and analyzed using ingenuity pathway analysis.

**Results:**

Microarray results showed that there were 1243 differentially expressed genes following treatment with CAL‐27 cells; 684 genes were upregulated and 559 were downregulated. Classical pathway analysis revealed a total of 89 signal transduction pathways with upregulated gene set enrichment, including lipopolysaccharide/interleukin (IL)‐1‐mediated inhibition of retinoid X receptor (RXR) function, agrin interactions at neuromuscular junctions, cholecystokinin/gastrin‐mediated signaling, toll‐like receptor signaling, and IL‐6 signaling. A total of 39 signal transduction pathways were enriched for the downregulated genes, including interferon signals, liver X receptor/RXR activation signals, and RhoGDI signals. In the disease and function analysis, the upregulated genes were enriched in viral infection, RNA virus replication, viral replication, cancer cell invasion, cell invasion, and other related functions, while downregulated genes were enriched in neuromuscular diseases, and leukocyte differentiation, antiviral response, connective tissue cell death and other functions.

**Conclusions:**

Gene chip analysis offers an effective means of screening differential gene expression between EVO‐treated TSCCs and controls, thus providing a sound basis for further research.

## 1 INTRODUCTION

Oral cancer is the most common malignant tumor in the head and neck, and accounts for 3% of all malignant tumors. The incidence and mortality rates of tongue squamous cell carcinoma (TSCC) are the highest among oral and maxillofacial squamous cell carcinomas, accounting for around one‐third of all oral cancer cases.^1^ The main methods currently used to treat TSCC include surgery, radiation, and chemotherapy. Because of the numerous drawbacks associated with surgical treatment and radiotherapy, chemotherapy has emerged as the most appropriate treatment for TSCC and has attracted increasing attention in this regard.^2^ Conventional chemotherapy drugs are associated with several disadvantages, including poor targeting, severe side effects, and adverse reactions. Therefore, identifying low‐cost chemotherapy drugs with high therapeutic efficacy and low toxicity has recently become a focal point of research on treatment of TSCC.

Evodiamine (EVO) is an indole quinazoline alkaloid extracted from the nearly ripe fruits of *Tetradium ruticarpum*. Its main pharmacological activities include stomach‐strengthening, analgesic, antiemetic, and diuretic effects, uterine contraction, vasodilation, and inhibition of *Escherichia coli*.^3^ EVO has been shown to induce apoptosis of tumor cells, and to inhibit angiogenesis, invasion, metastasis, and multidrug resistance in various tumors, including osteosarcoma,^4^ colon cancer,^5^ esophageal cancer,^6^ lung cancer,^7^ gastric cancer,^8^ and breast cancer.^9^ Guo et al^10^ demonstrated that EVO could inhibit the proliferation of hepatocellular cancer cells and significantly induce apoptosis by suppressing NOD1 signaling pathway. And Zhu et al^11^ showed that the mechanism of EVO against cholangiocarcinoma was likely due to the inhibition of interleukin (IL)‐6/STAT3 signaling with upregulating the expression levels of SHP‐2. Furthermore, increasing evidence demonstrates that EVO had an inhibitory effect on cancer cells through inducing intracellular calcium/JNK signaling‐mediated autophagy, inhibiting p‐nuclear factor‐κB, PI3K/AKT, and extracellular signal‐regulated kinase signaling pathway.^12^, ^13^, ^14^, ^15^ Previous studies conducted by our research group showed that EVO can inhibit proliferation and metastasis of the TSCC cell lines Tca8113 and CAl‐27, and promote their apoptosis. However, the molecular mechanisms by which EVO treats TSCC, the elucidation of which is required to identify new targets in the treatment of TSCC, remain unclear.

Biochip technology is a comprehensive, high‐tech modality that has emerged in recent years and is characterized by high parallelism, diversity, miniaturization, and automation. It includes gene chip, protein chip, cell chip, tissue chip, component microarray chip, channel microarray chip, and biosensor chip analyses.^16^ Gene chip analysis offers a robust strategy for identifying differential gene expression, underlying pathways, and cellular functions following drug treatment. In this study, we aimed to identify differentially expressed genes following EVO treatment of TSCC cell line CAL‐27, and to investigate the relationship between the differentially expressed genes and the EVO treatment, as well as the relationships among the genes themselves. Correlation analysis revealed the molecular mechanisms by which EVO affects TSCC and points to a potential novel target for clinical diagnosis and treatment.

## 2 MATERIALS AND METHODS

### 2.1 Cell line and culture

Human TSCC cell line CAL‐27 was cultured from CRL‐2095 of the American Type Culture Collection (Manassas, VA) in high‐glucose Dulbecco modified Eagle's medium containing 10% calf serum, 100 U/mL penicillin, and 100 U/mL streptomycin in a wet environment (5% CO_2_ at 37°C). Logarithmic growth phase cells were analyzed.

### 2.2 Total RNA extraction and determination

The TRIzol method was used to extract total RNA from the samples. The total extracted RNA was examined using NanoDrop 2000 (Thermo Fisher Scientific, Waltham, MA) and Bioanalyzer 2100 (Agilent, Chaoyang District, Beijing, China) instruments. Qualified samples were included in the chip experiment.

### 2.3 Chip preparation and hybridization

The GeneChip PrimeView Human Gene Expression profile chip (Affymetrix) was operated at 100 K. The labeling of the probes, and in vitro transcription, hybridization, and dyeing of the chips were performed by the Shanghai Jikai Gene Company Limited. The chips were scanned using the GeneChip Scanner 3000. The collected data were analyzed by ingenuity pathway analysis (IPA), using an online integrated analysis tool (http://www.ingenuity.com).

### 2.4 Quantitative real‐time polymerase chain reaction validation

We subjected 10 µg of total RNA to DNaseI treatment with 1 U DNaseI (NEB, New England Biolabs, MA). The reaction was carried out at 37°C for 15 minutes followed by heat inactivation at 85°C for 5 seconds. We then used 2 µg of DNase‐treated RNA for cDNA synthesis with reverse transcriptase (Bio‐Rad, CA), in accordance with the manufacturer's protocol. Primers were designed for selected transcripts from the transcriptome database (Table 1), and real‐time polymerase chain reaction (PCR) was performed with SYBR Green I master mix (Takara, Dalian City, Liaoning Province, China) on the fluorescent quantitative PCR apparatus (Lightcycler480).

**Table 1:** Primer sequences

Primer	Sequence (5′‐3′)
SOCS2 forward	5′‐AGCTGGACCAACTAATCTTCG‐3′
SOCS2 reverse	5′‐GTCCGCTTATCCTTGCACATC‐3′
DUSP1 forward	5′‐GCAGTACCCCACTCTACGATC‐3′
DUSP1 reverse	5′‐TTGAACCAGGAGCTGATGTCT‐3′
TGFB2 forward	5′‐GCAGAGTTCAGAGTCTTTCGT‐3′
TGFB2 reverse	5′‐CAGCAGGGACAGTGTAAGC‐3′
FGFR3 forward	5′‐CCCAAATGGGAGCTGTCTCG‐3′
FGFR3 reverse	5′‐CCCGGTCCTTGTCAATGCC‐3′
MMP10 forward	5′‐TGCTCTGCCTATCCTCTGAGT‐3′
MMP10 reverse	5′‐TCACATCCTTTTCGAGGTTGTAG‐3′
STAT2 forward	5′‐AATCGAGCTACTGCTGCCAAA‐3′
STAT2 reverse	5′‐CAGCTGCCTCAGGTGAAAVAA‐3′
GAPDH forward	5′‐TGACTTCAACAGCGACACCCA‐3′
GAPDH reverse	5′‐CACCCTGTTGCTGTAGCCAAA‐3′

Abbreviations: DUSP1, dual‐specificity phosphatase 1; FGFR3, fibroblast growth factor receptor 3; GAPDH, glyceraldehyde 3‐phosphate dehydrogenase; MMP10, matrix metallopeptidase; SOCS2, suppressor of cytokine signaling 2; STAT2, signal transducer and activator of transcription 2; TGFB2, tumor growth factor beta2

### 2.5 Statistical analysis

The data were analyzed using the Student *t* test and are expressed as x ± s. The data were obtained from three parallel experimental groups.

## 3 RESULTS

### 3.1 Sample quality control

The A260/A280 ratio of six groups of RNA samples was between 1.7 and 2.2, which confirmed that there was no contamination and the samples were of sufficient purity. According to the manufacturer's RNA quality‐control standard, samples with values 2100 RNA integrity number (RIN; >7.0) and 28S/18S (>0.7) in Bioanalyzer 2100 analysis can be considered qualified. As shown in Table 2, the tested samples satisfied the requirements for the chip experiment.

**Table 2:** RNA quality‐control standard data for the tested samples

		Thermo NanoDrop 2000		2100 result		
Sample number	Sample name	Concentration, ng/μL	A260/A280	RIN	28S/18S	Result
N2389‐1	CAL‐27‐EVO	528.7	1.95	9.4	2	Qualified
N2389‐2	CAL‐27‐EVO	499.7	1.97	9.6	1.9	Qualified
N2389‐3	CAL‐27‐EVO	526	1.99	9.5	2	Qualified
N2390‐1	CAL‐27‐NC	576.8	1.98	9.6	2	Qualified
N2390‐2	CAL‐27‐NC	598.5	2	9.5	1.8	Qualified
N2390‐3	CAL‐27‐NC	558.7	1.96	9.6	1.9	Qualified

Abbreviations: EVO, evodiamine; NC, negative control; RIN, RNA integrity number.

### 3.2 Gene chip results

**Figure 1:**
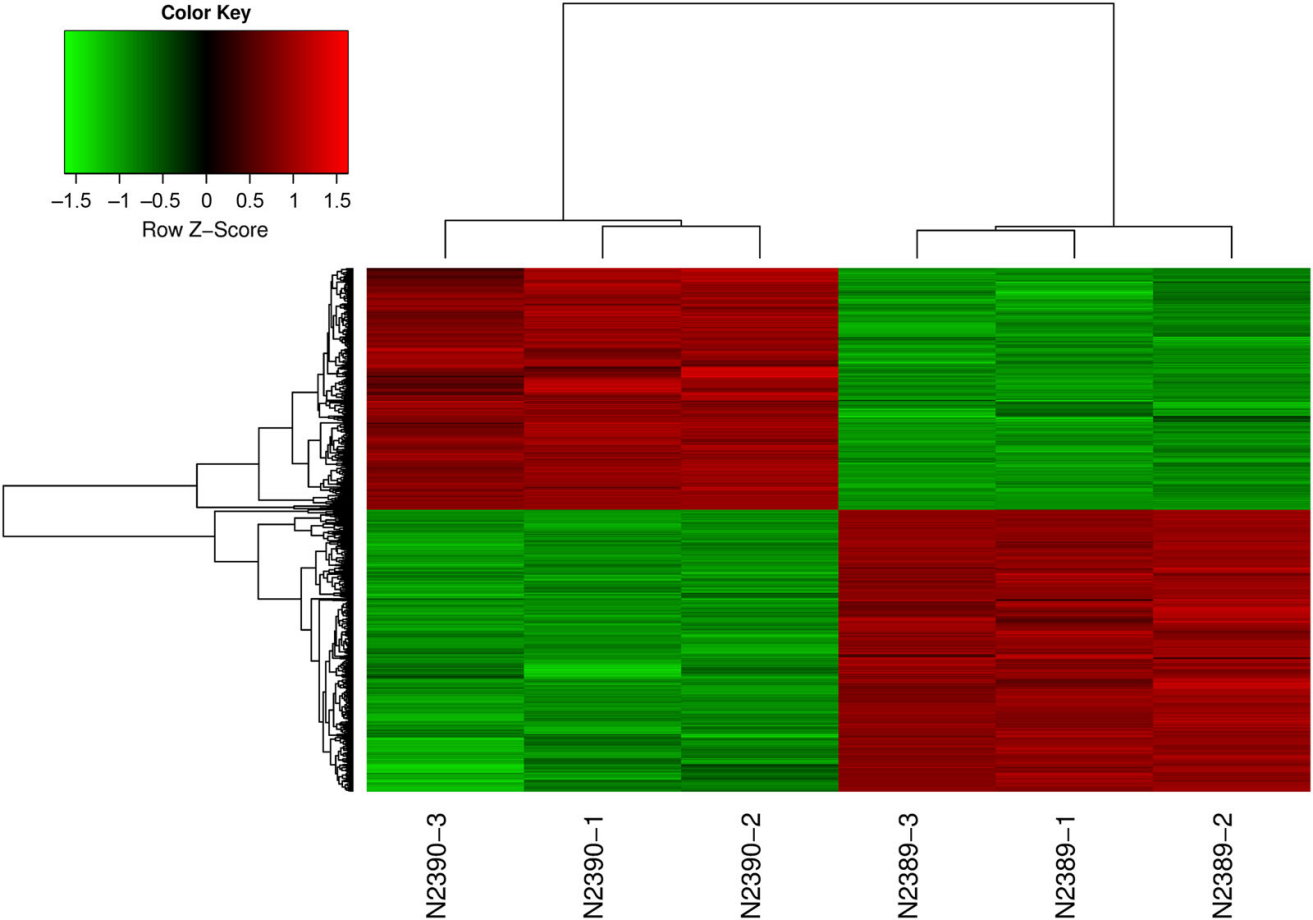
Cluster analysis of differentially expressed genes between the experimental and control groups

After the quality‐control and chip data had been standardized, *P* < 0.05 and ∣LogFC∣ ≥ 1.5 were set as the thresholds for determining differentially expressed genes. The gene chip results of the six groups of RNA samples showed that there were 1243 differentially expressed genes, including 684 upregulated and 559 downregulated genes. The most prominent upregulated gene was SERPINB2 (LogFC = 3.40), while the most obvious downregulated gene was matrix metallopeptidase 10 (MMP10) (LogFC = −3.76). The results are presented in Tables 3, 4, and scatter and cluster analyses of the 1243 differentially expressed genes are shown in Figures 1. Cluster maps show the differentially expressed genes; red color denotes upregulation (high expression level), green denotes downregulation (low expression level), black is moderate expression, and grey indicates no difference in expression between groups.

**Table 3:** Genes upregulated in the experimental group compared to the control group (top 10)

Gene symbol	Log fold change	*P* value
SERPINB2	3.3996162	1.59893E−07
SLC6A14	2.770502	1.20367E−05
MIG7	2.7265522	8.82098E−05
LOC100132240	2.7265522	8.82098E−05
SPRR1B	2.7095428	8.00705E−07
SPRR3	2.6802726	3.77358E−06
NT5E	2.6253428	3.43045E−05
AKAP12	2.5935268	1.39249E−06
ARL14	2.5877638	3.28356E−06
SPRR1A	2.5720496	1.6093E−06

**Table 4:** Genes downregulated in the experimental group compared to the control group (top 10)

Gene symbol	Log fold change	*P* value
MMP10	−3.760283	4.70275E−06
PTPRZ1	−3.7430208	2.79894E−05
MMP1	−3.7291782	3.49208E−06
MMP13	−3.5648968	7.70387E−07
TNFSF10	−3.3623605	8.19891E−07
MMP12	−3.0747347	0.000115643
CMPK2	−2.7681932	9.39619E−05
BCL11A	−2.6708074	7.93487E−06
DDX60	−2.6583266	7.40332E−06
OASL	−2.6263537	1.14295E−05

### 3.3 Classical pathway analysis

**Figure 2:**
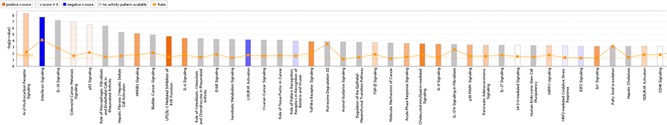
Signal pathway histogram showing the enrichment of differentially expressed genes in classical signaling pathways

**Figure 3:**
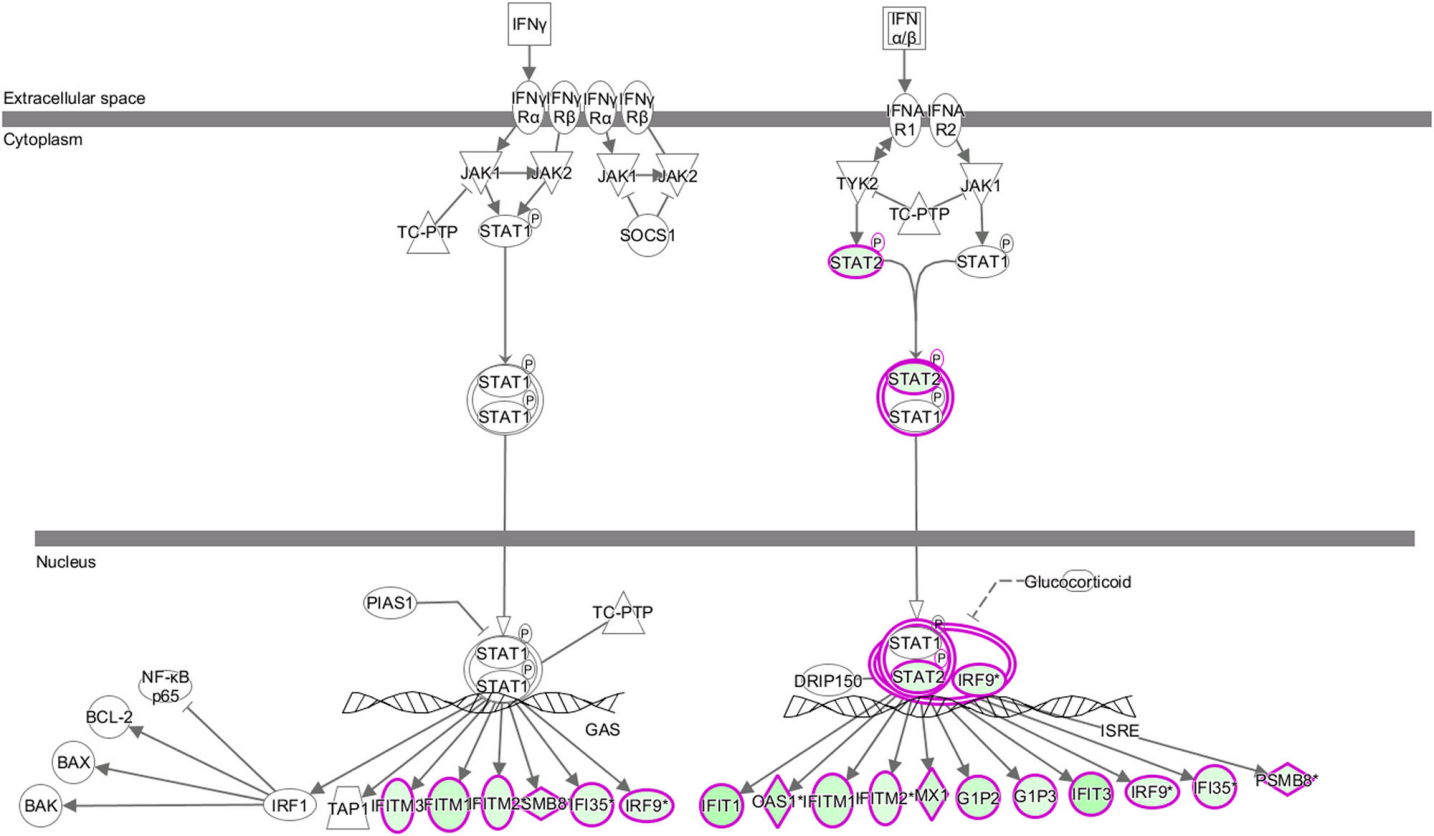
Experimental data predicting the pathways of interferon signaling inhibition. IFN, interferon; JAK, Janus kinase; NF‐κB, nuclear factor‐κB, STAT, signal transducer and activator of transcription

Classical IPA signaling pathways were analyzed for differentially expressed genes. All signaling pathways were sequenced by their log *P* value; *z* scores greater than zero indicate that the pathway is activated, while *z* scores less than zero indicate that the pathway is inhibited. The results showed that 89 pathways were activated; of these, lipopolysaccharide/IL ‐1‐mediated inhibition of retinoid X receptor (RXR) function, agrin interaction at the neuromuscular junction, cholecystokinin/gastrin‐mediated signaling, toll‐like receptor (TLR) signaling, and IL‐6 signaling were significantly activated, while 53 pathways were inhibited, of which interferon (IFN) interaction at the neuromuscular junction, cholecystokinin/gastrin‐mediated signaling, TLR signaling and IL‐6 signaling were significantly activated. N signaling, liver X receptor/RXR activation, RhoGDI signaling (Figure 2). The results showed that the expression levels of IFIT1, IFIT3, and signal transducer and activator of transcription 2 (STAT2) in the IFN signaling pathway were significantly downregulated following the treatment of CAL‐27 cells with EVO. The colors denote the expression levels: green represents downregulation and red upregulation. The hue relates to the magnitude of expression differences, and the purple‐red border separates different genes (Figure 3).

### 3.4 Disease and function analyses

**Figure 4:**
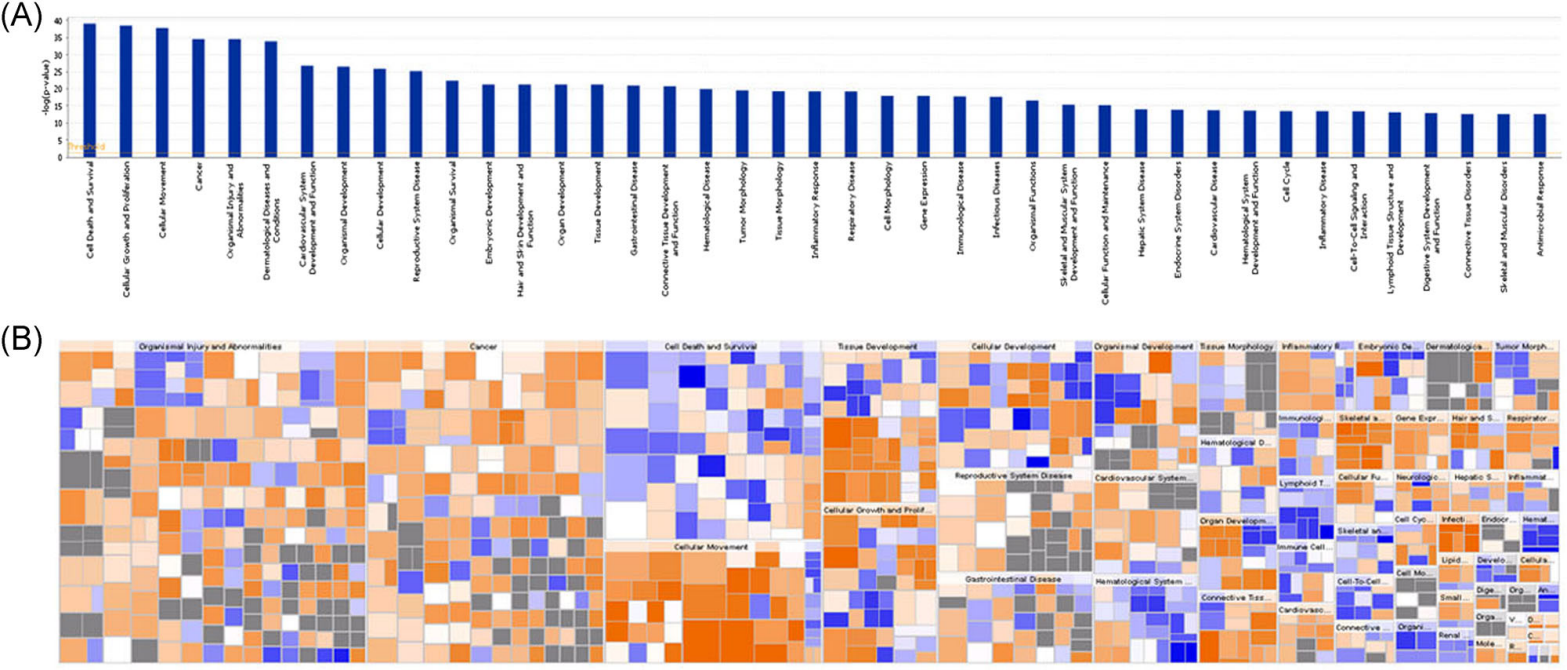
Disease and functional analyses of differentially expressed genes in the CAL‐27 after EVO treatment. A, Disease and function histogram showing the enrichment of differentially expressed genes in disease and function. B, Disease and functional thermograms showing the relationship between differential gene expression and function and disease

Enrichment of differentially expressed genes was sorted from high to low according to log *P* values. The results indicate that differential genes are mainly enriched in cell death and survival, cellular growth and proliferation, cellular movement, cancer, organismal injury and abnormalities, and dermatological diseases and conditions pathways, among others (Figure 4A). Disease and functional thermography (Figure 4B) show the relationship between differential gene expression and function and disease. The related functions of obvious activation were virus infection (*z* = 3.768), replication of RNA virus (*z* = 3.537), virus replication (*z* = 3.486), invasion of cancer cell lines (*z* = 3.208), cell invasion (*z* = 3.013), and related functions of obvious inhibition were neuromuscular diseases (*z* = −3.206). Leukocyte differentiation (*z* = −2.333), antiviral response (*z* = −2.246), connective tissue cell death (*z* = 2.233), among others.

### 3.5 Verification of gene chip results using real‐time fluorescence qPCR

**Figure 5:**
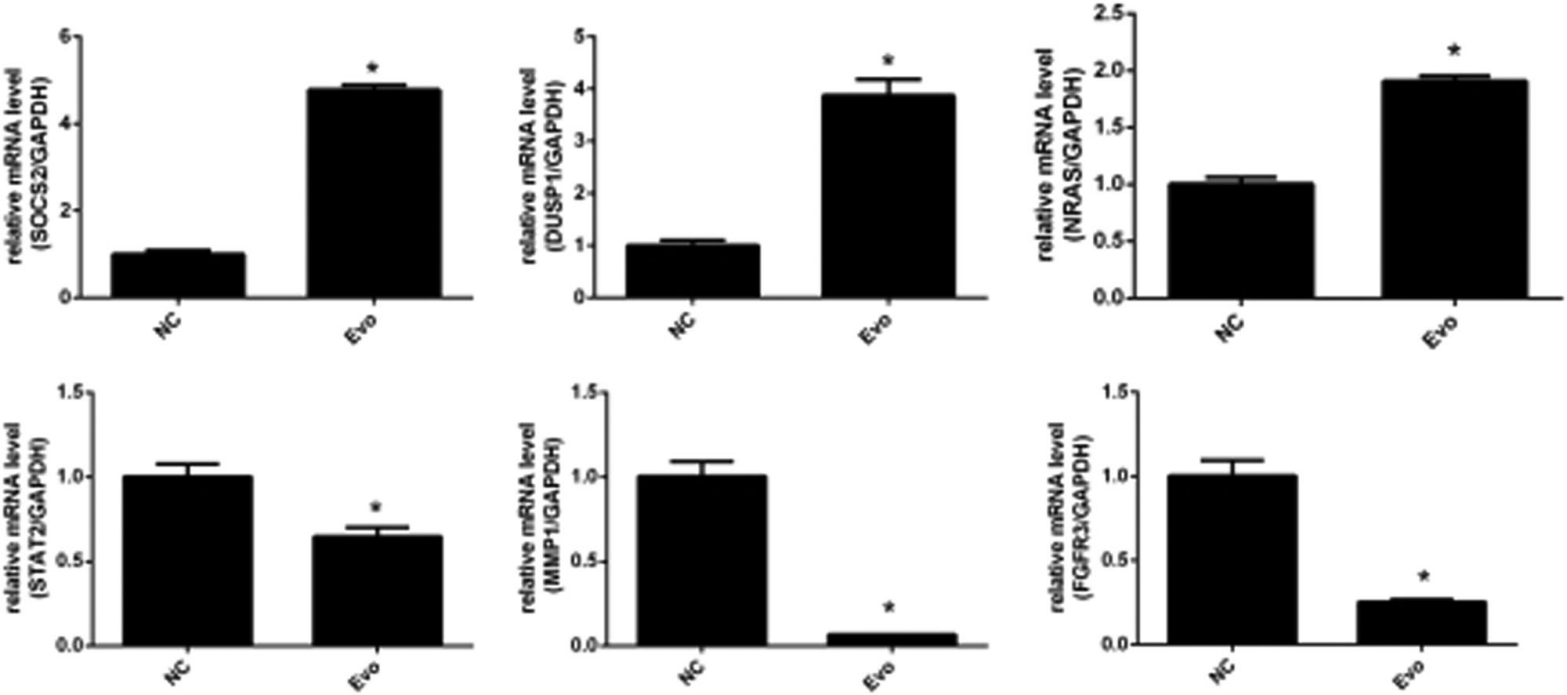
Fluorescence quantitative polymerase chain reaction analysis verified that the microarray results were accurate and reliable. DUSP1, dual‐specificity phosphatase 1; EVO, evodiamine; FGFR3, fibroblast growth factor receptor 3; GAPDH, glyceraldehyde 3‐phosphate dehydrogenase; MMP10, matrix metallopeptidase; NC, negative control; SOCS2, suppressor of cytokine signaling 2; STAT2, signal transducer and activator of transcription 2

Of the 1243 differentially expressed genes in the EVO‐treated CAL‐27 cells, 30 were randomly selected for qPCR analyses. The results showed that the expression levels of suppressor of cytokine signaling 2 (SOCS2), dual‐specificity phosphatase 1 (DUSP1), and NRAS were upregulated, while those of STAT2, MMP1, and FGFR3 were downregulated. Comparison with the microarray results allowed us to confirm that the differential gene expression results yielded by microarray and qPCR analyses were completely consistent, verifying that the microarray results are accurate and reliable (Figure 5).

## 4 DISCUSSION

TSCC is associated with early lymph node metastasis, high malignancy, a rapid growth rate, and the absence of early specific biomarkers, resulting in a 5‐year survival rate of only 50%.^17^, ^18^ With advances in medical technology and improved living standards, quality of life is being continuously enhanced. While improving the survival rate among cancer patients continues to be a priority, patients’ quality of life has become an important indicator for measuring the therapeutic effects of cancer treatment.^19^ The research on traditional Chinese medicine (TCM) is extensive and profound. For cancer treatment, TCM has the advantages of being cost‐efficient, readily accessible, and applicable to a wide range of targets with low toxicity and minimal side effects.^20^ The anticancer properties of EVO have been demonstrated in several studies. Our experimental use of EVO for treating TSCC corroborates these findings, while our use gene chip technology results have pointed to new targets for the treatment of TSCC.

Following EVO treatment of the CAL‐27 cell line, we analyzed the classical signaling pathways of differentially expressed genes, and found the most obvious inhibition in the IFN signaling pathway (ie, highest *z* score at *P* < 0.05). IFN type is classified based on the receptors involved in signal transmission and is typically divided into type I (IFN‐α, ‐β, ‐κ, ‐ε, and ‐ω), type II (IFN‐γ), and type III (including various subtypes of IFN‐λ). The most important classical signaling pathway mediated by IFN is the Janus kinase (JAK)/STAT pathway,^21^ which is among the most important pathways of cytokine signal transduction and is instrumental not only in inflammatory responses, but also in cell proliferation and differentiation, apoptosis, and immune regulation.^22^ IFNα/β is secreted from the cell exterior, where it binds to interferon receptors (IFNARs) on the cell surface to activate the downstream JAK/STAT signaling pathway; this leads to the phosphorylation of STAT1/STAT2. p‐STAT1/p‐STAT2 binds with IRF‐9 to form the interferon‐stimulating gene factor 3 complex, which is transferred to the nucleus and initiates the transcription of the corresponding binding sites.^23^ Studies have increasingly shown that abnormal activation of the IFN signaling pathway can promote cell growth and the cell cycle, and inhibit apoptosis. This may be closely related to the development of tumors.

The SOCS family are negative regulators produced by cells and feedback mechanisms that block cytokine signal transduction.^24^ SOCS regulates the JAK/STAT signaling pathway through competitive binding with JAK, the blocking of STAT‐binding sites, or promotion of ubiquitin‐mediated degradation of JAK and STAT proteins.^25^ The SOCS family consists of eight members: SOCS 1 to 7 and cytokine‐inducible SH2‐containing protein, which are characterized by a variable N‐terminal region and SH2 domain, and a highly conserved C‐terminal SOCS box.^26^ The SH2 domain and SOCS box can attenuate the signal transduction associated with JAK or cytokine receptors.^26^ The structure of SOCS2 corresponds to the basic structure of the SOCS family members.^27^ Unlike SOCS1 and SOCS3, SOCS2 cannot directly inhibit JAK activity, but it can inhibit STAT activation by competitively binding in the SH2 region, thus inhibiting the JAK/STAT pathway.^28^ SOCS2 is key in regulating metabolism,^29^ bone growth,^30^ tumorigenesis,^31^ and immunity.^32^ However, the relationship between SOCS2 and TSCC remains under‐researched. We found that EVO can upregulate the expression of SOCS2 and downregulate the expression of STAT2 in tongue Cal‐27 cells. The mechanisms underlying the anticancer activity of EVO may involve its ability to efficiently inhibit further activation of STAT2 by upregulating the expression of SOCS2, and then inhibiting the activity of the JAK/STAT pathway through negative feedback mechanisms.

In this study, we screened out abnormally expressed genes in rutaecarpine‐treated TSCC using gene chip technology. The anticancer activity of EVO results from the interaction between and regulation of multiple targets. Further study and analysis of these targets will further our understanding of the mechanisms underlying its anticancer properties at the molecular level and will provide new directions for treating TSCC and exploring gene therapy.

## CONFLICT OF INTERESTS

The authors declare that there are no conflict of interests.

## AUTHOR CONTRIBUTIONS

JW and JC conceived and designed the experiments. YW and JW performed the experiments. YW, JZ, and JC analyzed the data. YW, YZ, and YS interpreted the results. YW and JW wrote the paper. All authors read the manuscript drafts, contributed edits, and approved the final manuscript.
